# Publisher Correction: Identification of a peptide recognizing cerebrovascular changes in mouse models of Alzheimer’s disease

**DOI:** 10.1038/s41467-018-03517-0

**Published:** 2018-03-09

**Authors:** Aman P. Mann, Pablo Scodeller, Sazid Hussain, Gary B. Braun, Tarmo Mölder, Kadri Toome, Rajesh Ambasudhan, Tambet Teesalu, Stuart A. Lipton, Erkki Ruoslahti

**Affiliations:** 1 Cancer Research Center, Sanford Burnham Prebys Medical Discovery Institute, La Jolla, CA 92037 USA; 2 Laboratory of Cancer Biology, Institute of Biomedicine and Translational Medicine, University of Tartu, Tartu, 50411 Estonia; 3 AivoCode Inc, La Jolla, CA 92037 USA; 4 Neurodegenerative Disease Center, Scintillon Institute, San Diego, CA 92121 USA; 5 Department of Neurosciences, University of California, San Diego, School of Medicine, La Jolla, CA 92093 USA; 6 Center for Nanomedicine and Department of Cell, Molecular and Developmental Biology, University of California, Santa Barbara, Santa Barbara, CA 93106 USA

Correction to: *Nature Communications* 10.1038/s41467-017-01096-0, published online 10 November 2017.

The original version of the Supplementary Information associated with this Article inadvertently omitted Supplementary Table 1. The HTML has now been updated to include a corrected version of the Supplementary Information.

