# Supplementary material for: Identification of a peptide recognizing cerebrovascular changes in mouse models of Alzheimer’s disease
Source: Nat Commun. 2017 Nov 10;8:1403. doi: 10.1038/s41467-017-01096-0 (PMC5680235; doi:10.1038/s41467-017-01096-0)
Supplement: Supplementary file 1 — Supplementary Information [file 41467_2017_1096_MOESM1_ESM.pdf]

SUPPLEMENTARY INFORMATION

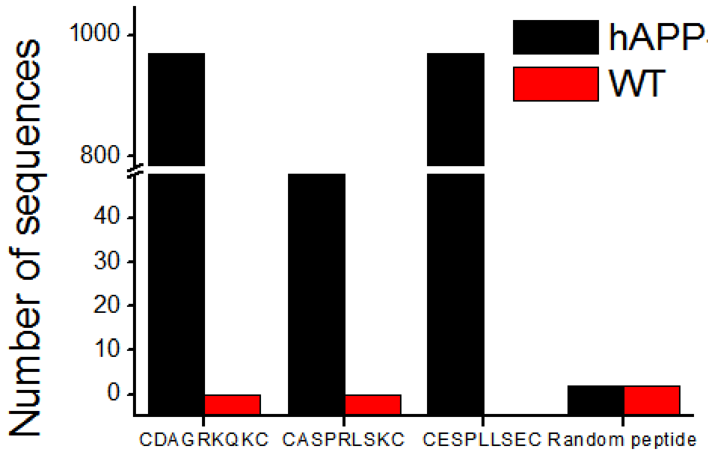

S1. Peptide sequences recovered from the hippocampus in phage library screening. Cyclic CX7C library was intravenously injected to hAPP-J20 mice and wild-type (WT) littermate mice at 9 months of age. After 30 min, the mice were perfused and phages were recovered from the hippocampus. Phage insert DNA was subjected to high-throughput sequencing. Comparison of the number of the most common insert sequences in the phage pools recovered from the hAPP-J20 and WT mice is shown.

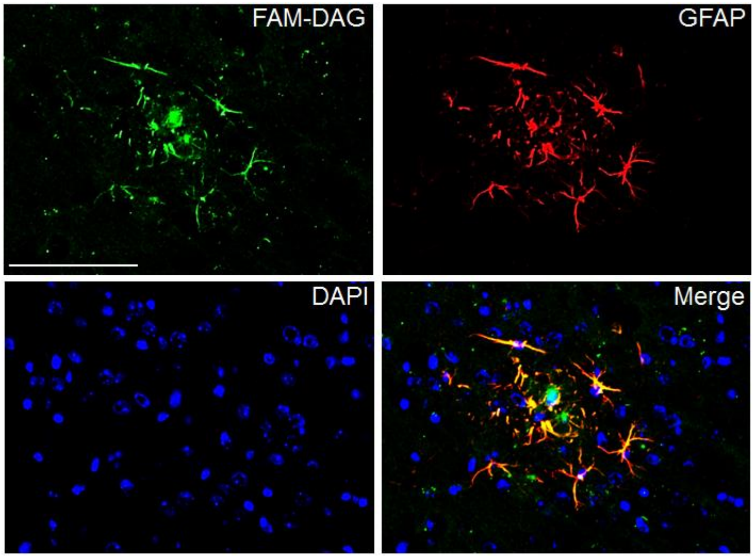

S2. DAG homing to the cortex of 9month old hAPP-J20. Immunofluorescence analysis on frozen coronal sections from DAG injected hAPP-J20 (9 month old) brain showing the cortex stained for FAM (green), and counterstained with DAPI (blue). Scale bar, 100 µm.

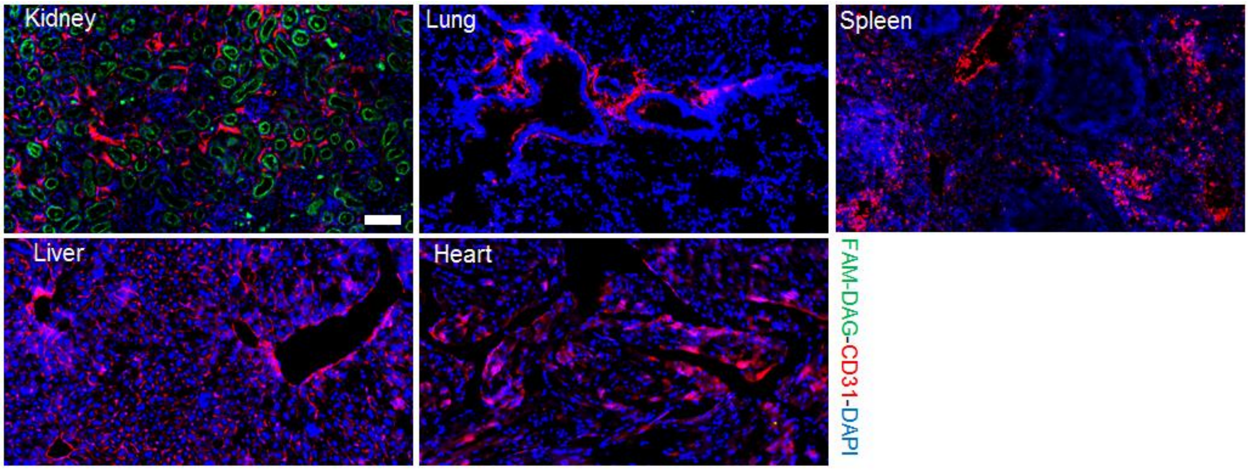

S3. DAG does not accumulate in vessels of organs other than the brain. FAM-DAG was intravenously injected into 9-month old hAPP-J20 mice and allowed to circulate for 30 min, after which the mice were perfused, the organs were fixed, sectioned and stained for FAM (green), CD31 (red) and counterstained with DAPI (blue). Scale bar, 50μm.

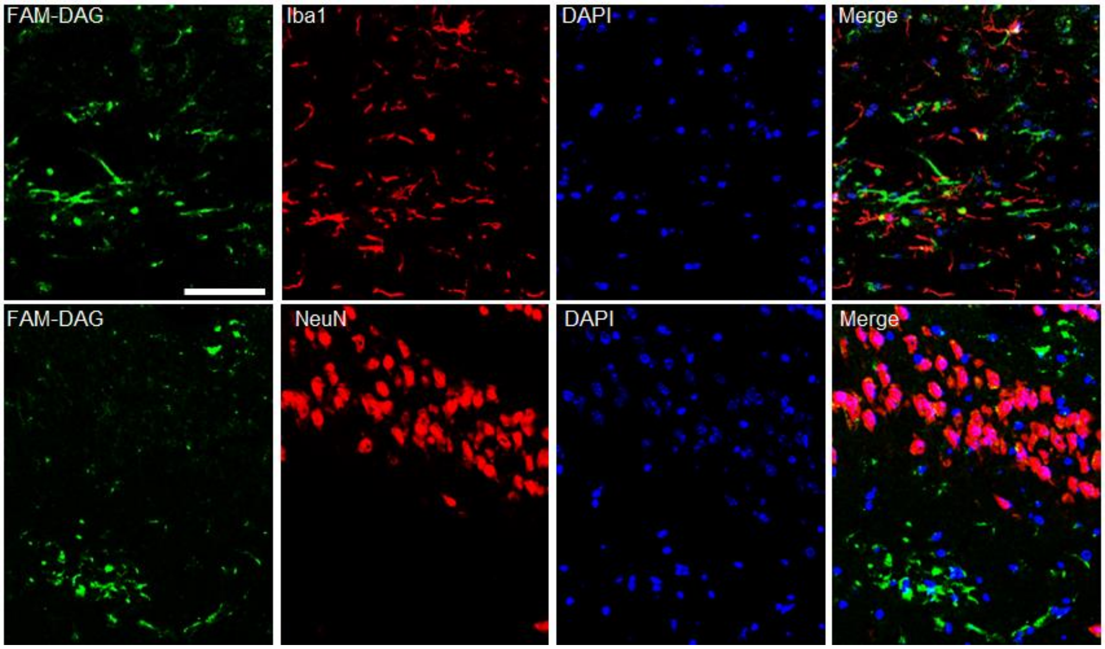

S4. DAG does not co-localize with microglia or neuronal cells in AD. Immunofluorescence on frozen coronal sections from hAPP-J20 (9 month old) brain showing the hippocampus stained for Iba-1, and NeuN (shown in red), and for FAM-DAG (green) using anti-fluorescein antibody and counterstained with DAPI (blue). Scale bar – 50 μm.

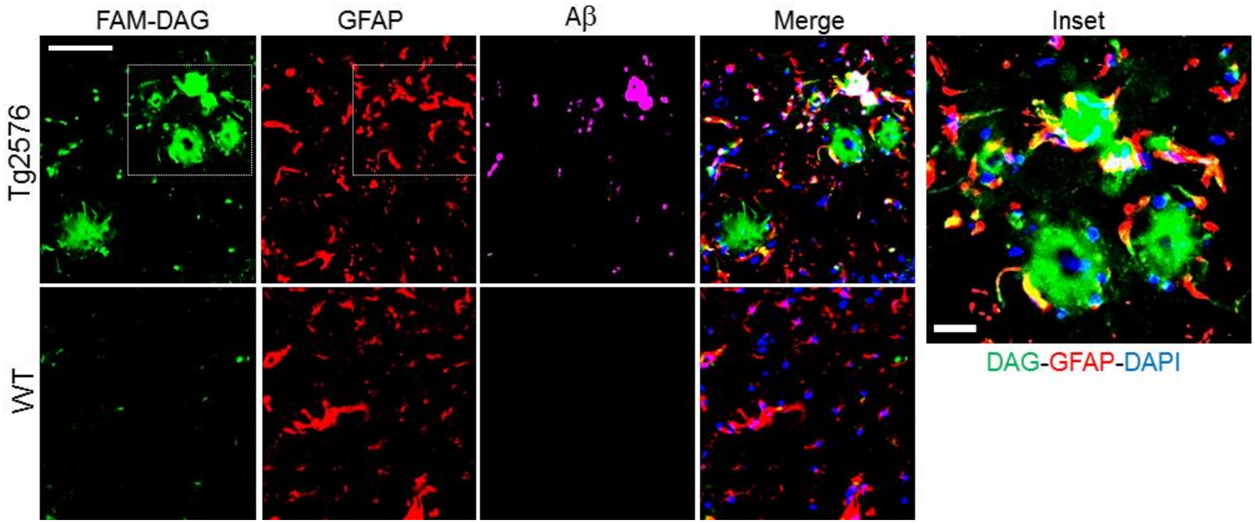

S5. DAG targets astrocytes surrounding Aβ plaques in the Tg2576 mice. FAM-DAG was intravenously injected into 20-month old Tg2576 and WT mice and allowed to circulate for 30 min, after which the mice were perfused, the brains were fixed, sectioned and stained for FAM (green), GFAP (red), Aβ (magenta) and counterstained with DAPI (blue). The region shown is the hippocampus. The inset shows a higher magnification view from the merge panel. Scale bars, 50µm, 20µm (inset).

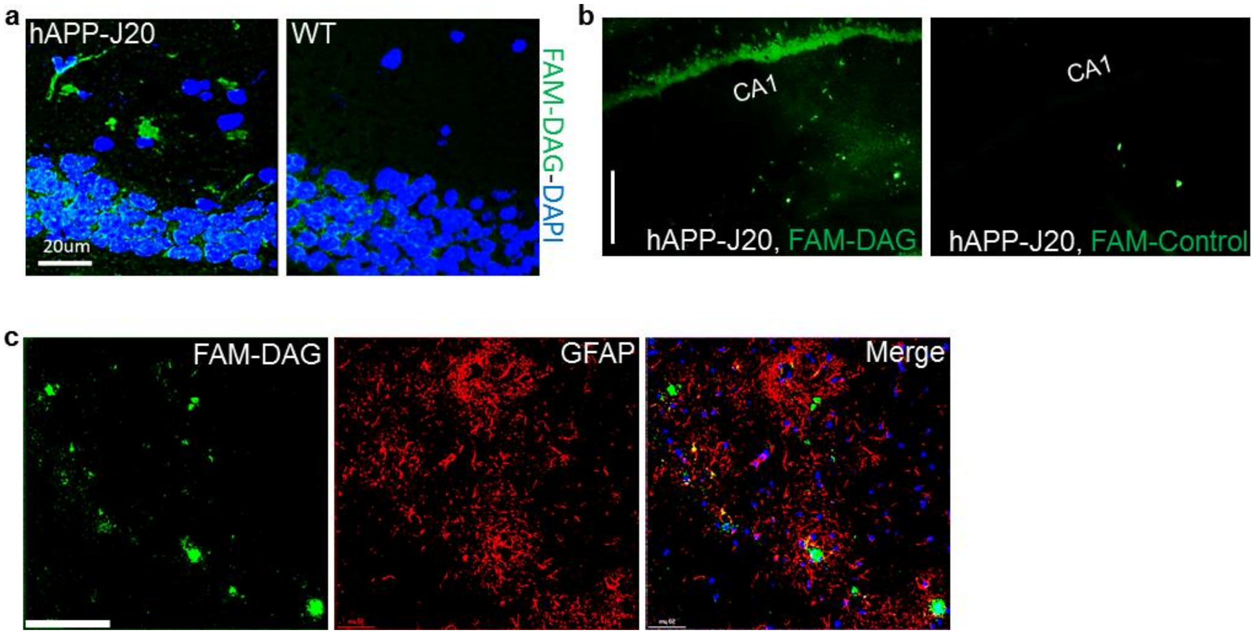

S6. DAG binds to brain sections from hAPP-J20 mice. Representative immunofluorescence images from *ex vivo* binding experiment on brain sections from 9-month old hAPP-J20 and WT mice incubated with FAM-labeled DAG **(a)** and comparison of FAM-labeled DAG and FAM-labeled control peptide incubated on sections from hAPP-J20 mice **(b)**. Sections were counterstained with DAPI. **(c)** DAG-incubated sections from panel **a** were stained for GFAP (red) and counterstained with DAPI (blue). The region shown is the hippocampus. Scale bars, 20µm (panel A), 100 µm (panels B and C).

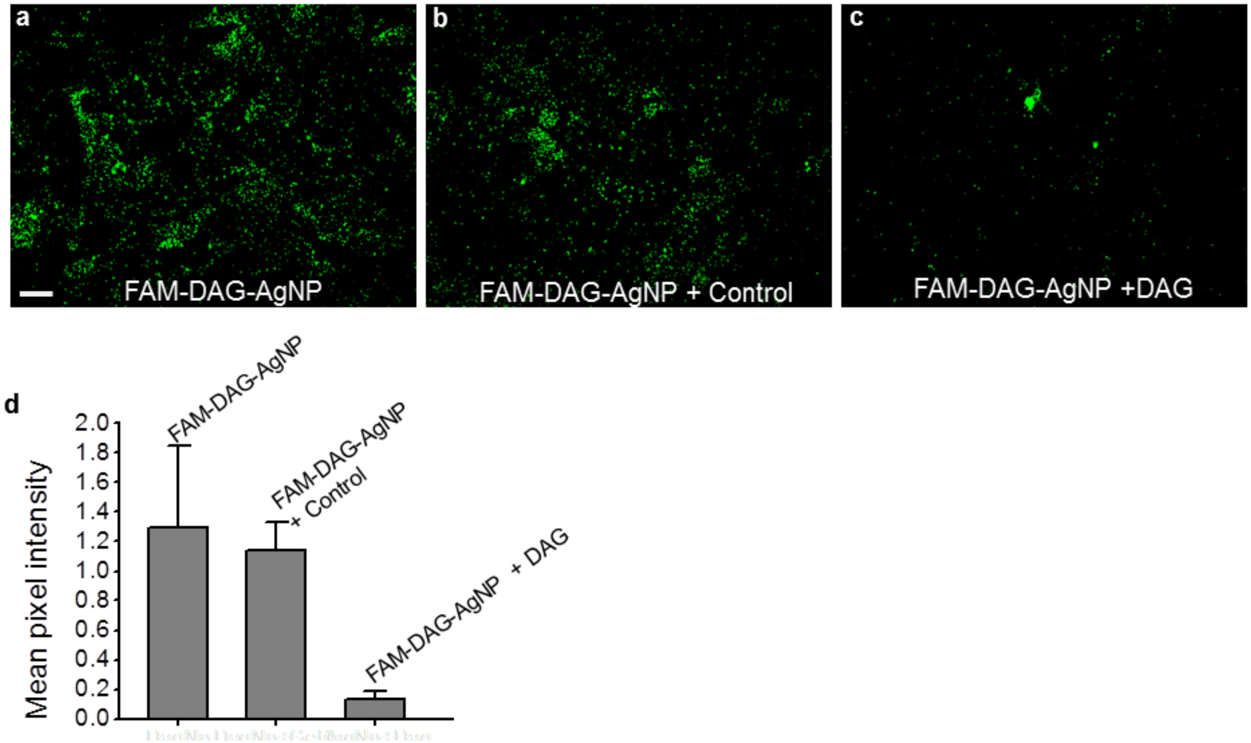

S7. DAG-coated silver nanoparticles (DAG-AgNPs) bind to human astrocytoma U251 cells. Cultured U251 cells were incubated with DAG-AgNPs alone **(a)**, in the presence of free non-labeled control peptide **(b)**, or an excess of free, non-labeled DAG **(c)** (both at 200μM), for 1 hour at 37°C. Nanoparticle binding was quantified from fluorescence micrographs using ImageJ software **(d)**. Scale bar, 20μm.

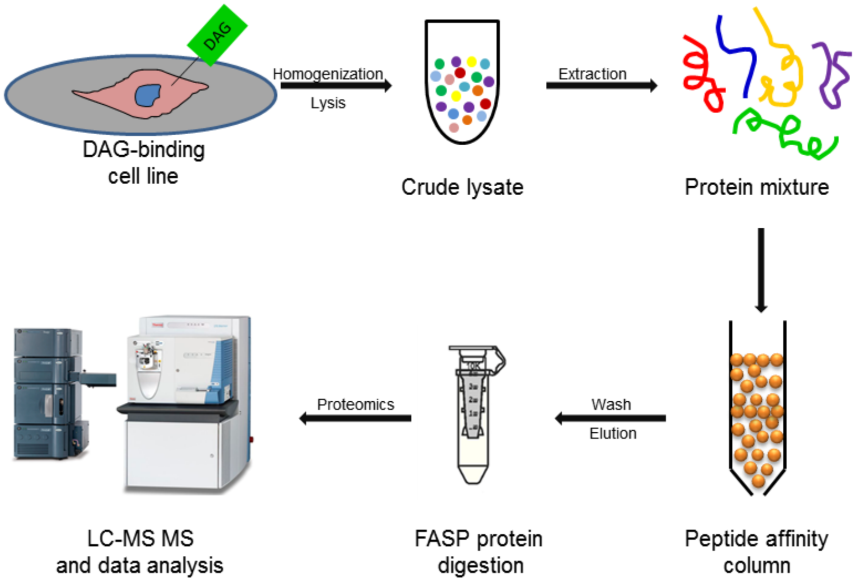

S8. Schematic for identification of DAG receptor from a DAG-binding cell line. See methods section for details.

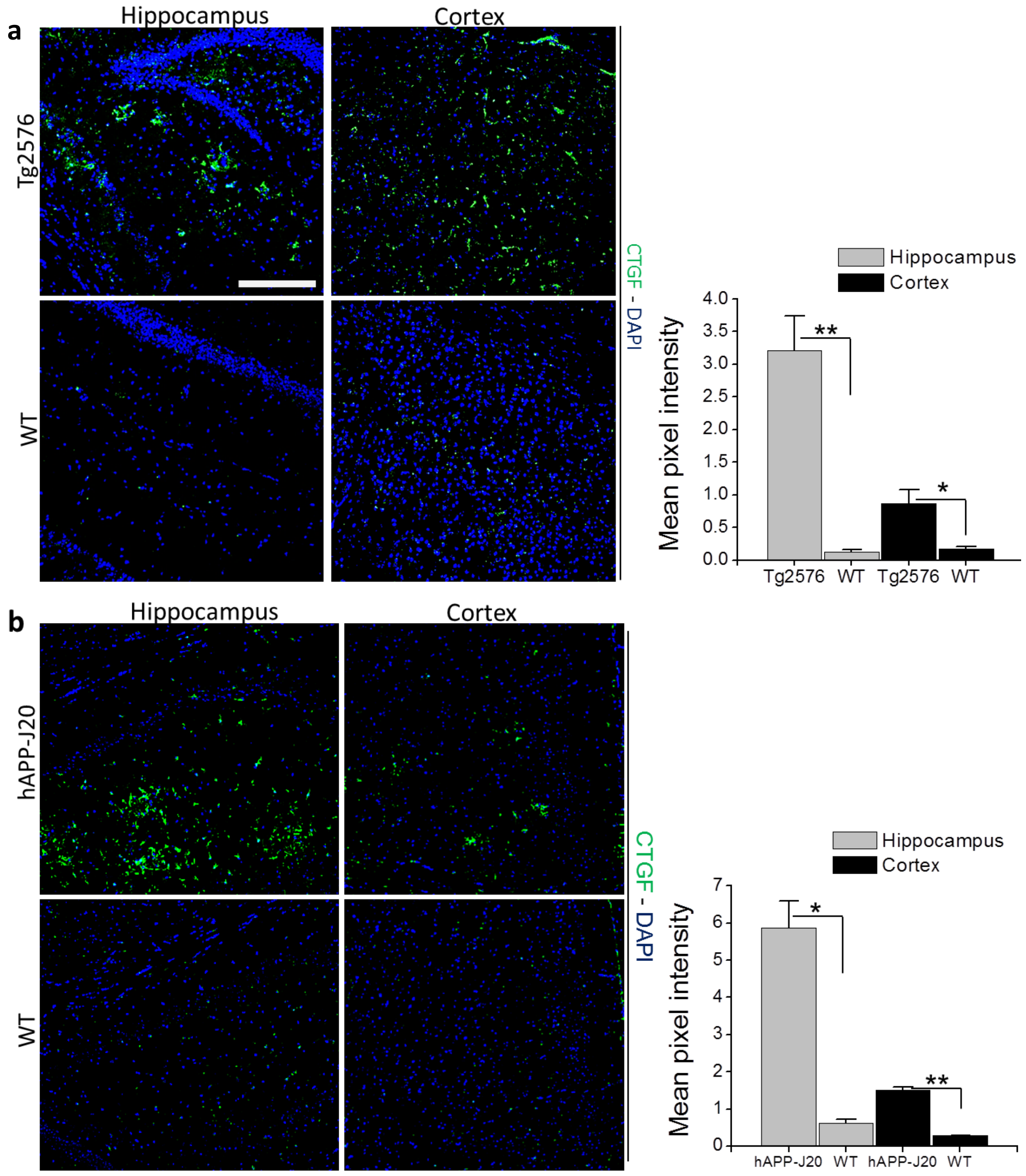

S9. CTGF is overexpressed in mouse models of AD. Frozen coronal brain sections from Tg2576 and WT mice **(A)** and hAPP-J20 and WT mice **(B)** were stained for CTGF (green) and counterstained with DAPI. Green signal was analyzed by fluorescence microscopy and quantified from the hippocampus and cortex regions of the Tg and the WT mice. Scale bar: 200µm. *P<0.05, **P<0.01.

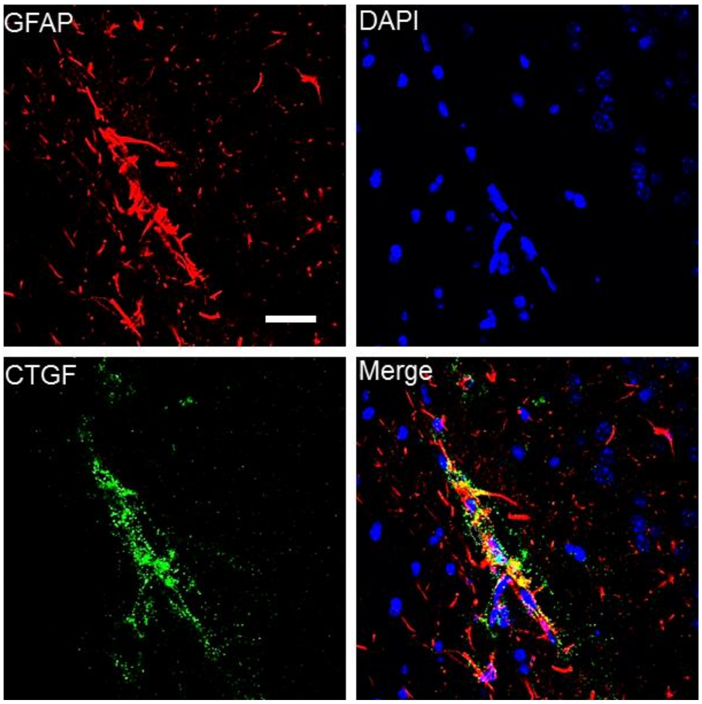

S10. CTGF is associated with astrocytes in brains of hAPP-J20 mice. Representative immunofluorescence images show strong immunoreactivity for CTGF in close association with GFAP+ cells in the hippocampus of hAPP-J20 mice. The mice were perfused, and the brains were fixed, sectioned, and stained for CTGF (green), GFAP (red) and counterstained with DAPI. Scale bar, 20µm.

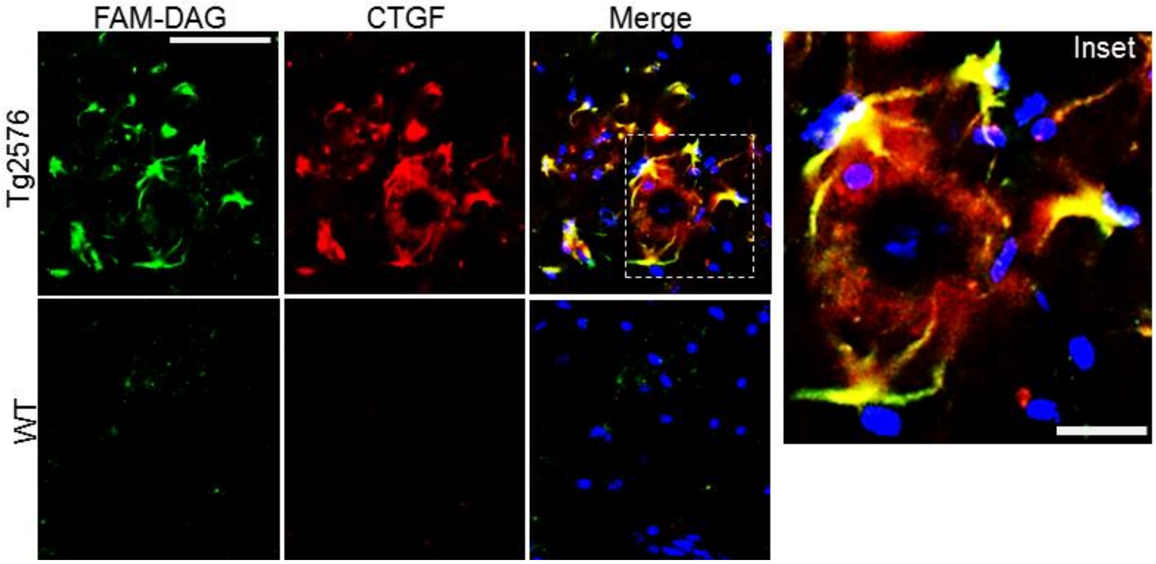

S11. DAG colocalizes with CTGF in the Tg2576 model. DAG was injected intravenously to 20-month-old Tg2576 and WT mice and allowed to circulate for 30 mins, mice brains were sectioned and stained for FAM (green) and CTGF (red). The region shown is the hippocampus. Scale bar: 50µm, 20µm (inset).

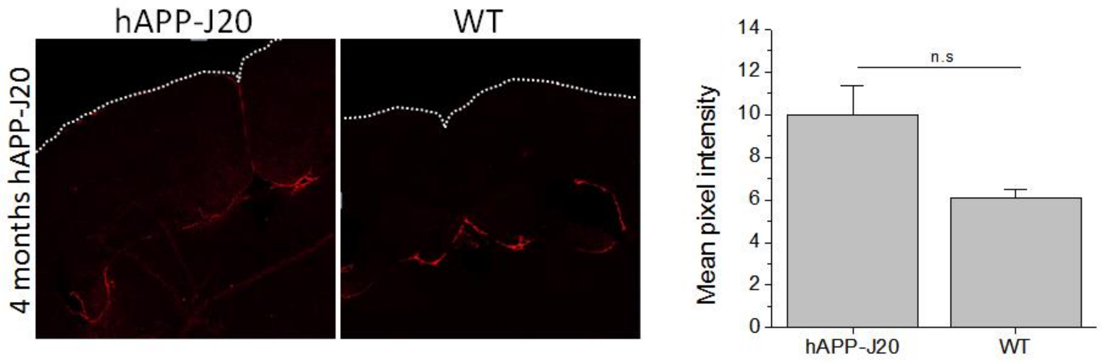

S12. Blood brain barrier leakage in 4-month-old hAPP-J20 mice. Frozen coronal brain sections from 4 month-old hAPP-J20 mice and age matched WT controls were stained for mouse IgG (red). Total fluorescence intensity was quantified and plotted. (Mean ± SEM, *n*=3, ns – not significant).

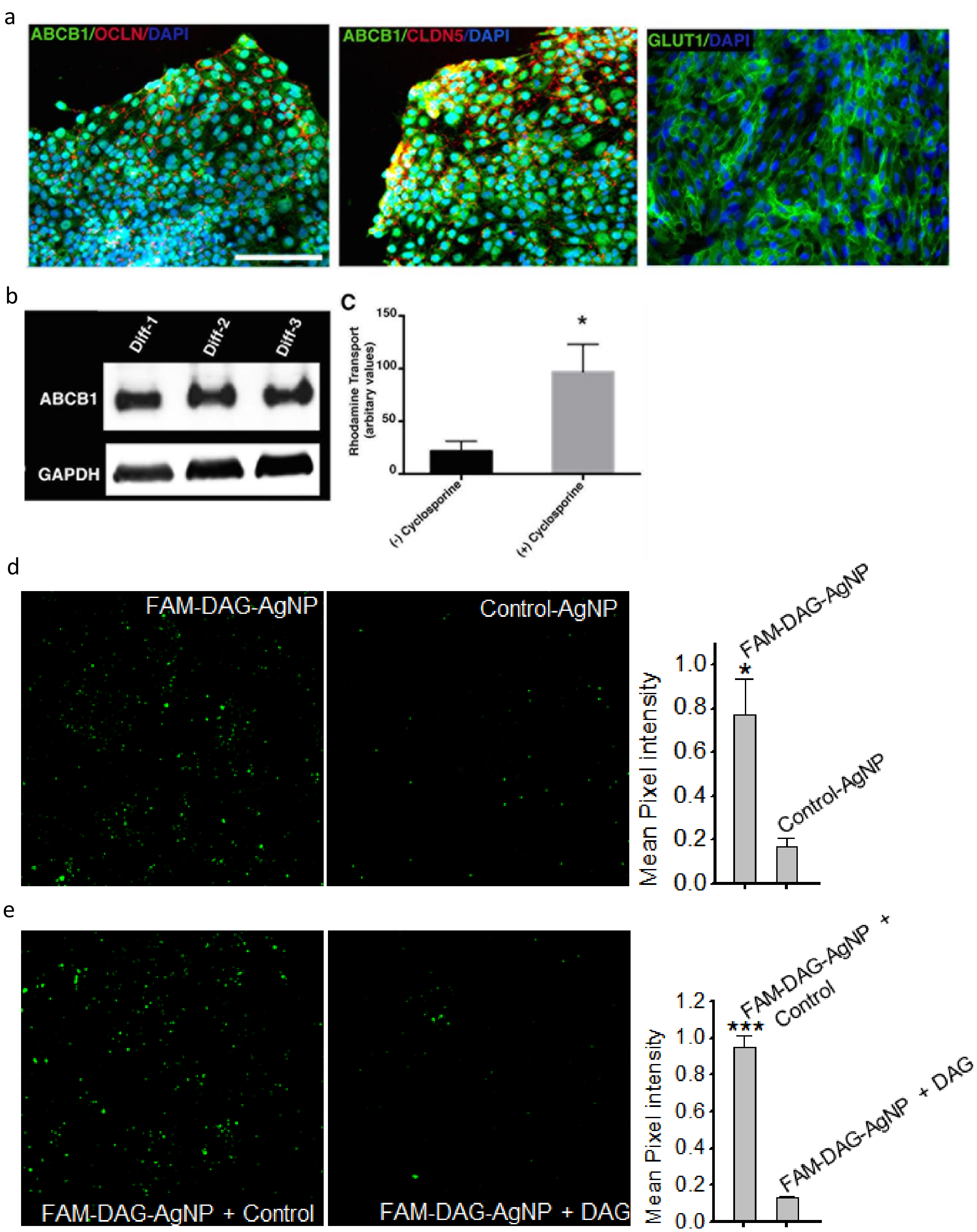

S13. DAG-AgNPs bind to cells derived from AD patient derived iPS cells. (a-c) Characterization of hiPSC-derived BMECs: hiPSCs were seeded on matrigel-coated plates and differentiated in unconditioned medium for 7 days, followed by culture in EC medium for another 5 days. The cells were then sub-cultured and expanded on collagen/fibronectin substrate for at least one week before characterization to confirm their BMEC identity. (a) Representative immunohistograms showing hiPSC-derived BMECs displaying immunoreactivity for BMEC markers. Co-expression of the efflux transporter p-glycoprotein (ABCB1; green) with the tight junction proteins occludin (red; left panel) and claudin-5 (red; middle panel). Right panel, expression of BMEC glucose transporter Glut-1 (green). DAPI-labeled nuclei are shown in blue in all panels. (b) Immunblot analysis of BMECs from three

independent experiments differentiating hiPSCs (designated Diff-1, Diff-2, Diff-3). Cells display expression of the BMEC marker p-glycoprotein (ABCB1). GAPDH protein served as a loading control. (c) Transwell assay to evaluate functional expression of efflux transporters in BMECs. BMECs were co-cultured with astrocytes in dual chamber Transwell plates, with BMECs seeded on the upper (apical) chamber and astrocytes seeded on the lower (basolateral) chamber. Cyclosporin A, a p-glycoprotein inhibitor, was added to the upper chamber in a subset of the samples and incubated for 30 min. All samples were then incubated with Rhodamine 123 for an additional 60 min after which fluorescence in the astrocytes lysates was measured using a plate reader. Note increased transport of Rhodamine 123 to the basolateral chamber in the samples receiving Cyclosporin A, indicating the presence of a functional ABCB1 efflux transporter. Error bars indicate SEM (n =3 ; *P < 0.1 by Student's t-test). **(d-e) AgNP binding to iPSc derived BMECs**. Overlay binding assay was performed on BMECs by incubating AgNPs coated with FAM-DAG **(d, left panel)** or FAM-labeled control peptide **(d, right panel). (e).** The cells were incubated with DAG-AgNPs together with 200µM of free, non-labeled control peptide **(left panel)** or non-labeled DAG **(right panel).** Fluorescent microscopy analysis of native FAM fluorescence (green) was used to observe AgNP binding, and the binding was quantified using ImageJ software (bar graphs). Free DAG peptide significantly inhibits the binding of the AgNPs, indicating specificity of the binding. ***P<0.001.

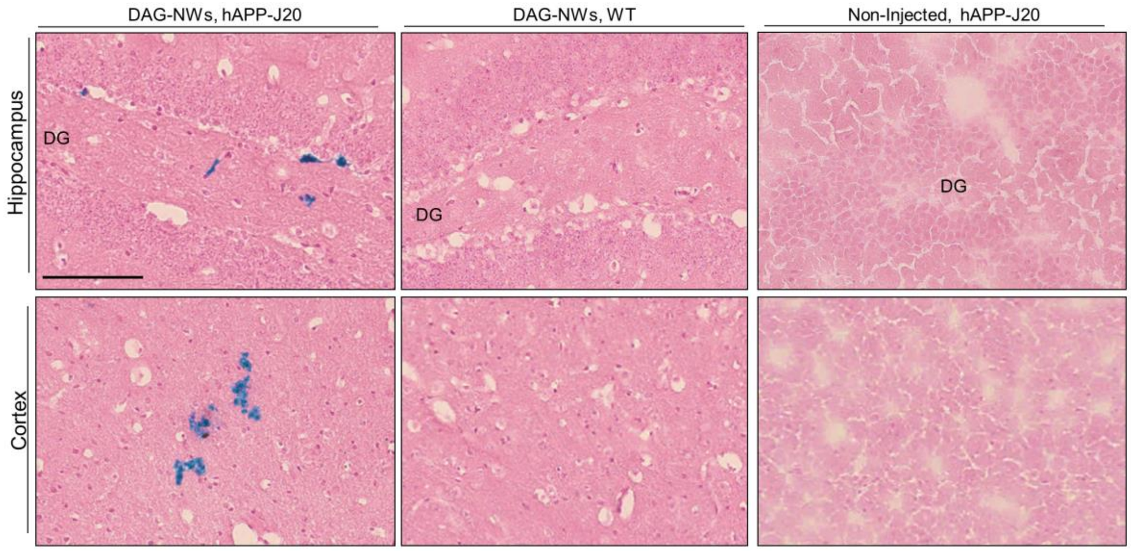

S14. DAG mediated delivery of nanoparticles to AD brain. Iron oxide nanoparticles (NWs) conjugated with DAG (DAG-IONWs) were injected i.v. into 9 month-old hPPP-J20 mice and allowed to circulate for 5 hours before perfusion (n=2). In the brain sections shown, NWs were visualized by Prussian blue staining and the sections were counterstained with nuclear fast red. Shown are representative sections with DAG-IONWs in the hippocampus (top panel) and cortex (bottom panel). The controls include DAG-IONWs injected into age-matched WT mice and non-injected hPP-J20 mice. DG*:* dentate gyrus. Scale bar – 100 µm.

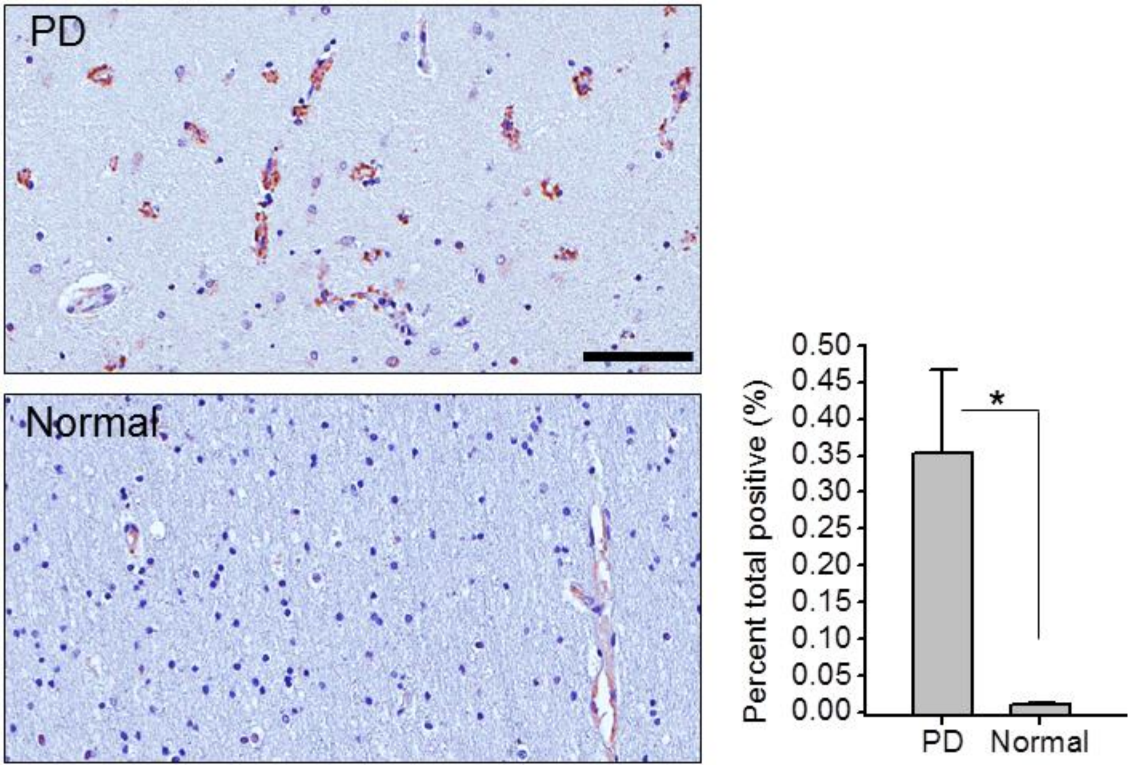

S15. CTGF is overexpressed in human PD brain. Representative immunohistochemical images of CTGF expression in brain sections from a human PD brain (*top*) and a normal human brain (*bottom*) show significantly elevated CTGF in only in the PD patient brain when quantified (*at right*). Scale bar, 100 µm. *$P < 0.05$.

**TABLE 1:**<br>**Proteins identified by DAG peptide-affinity chromatography and mass spectrometry (MS) analysis**		
**Protein Name**	**UniProt ID**	**Gene Name**
Profilin-2	P35080	PFN2
Antileukoproteinase	P03973	SLPI
Connective tissue growth factor	P29279	CTGF
Brain-specific angiogenesis inhibitor 1-associated protein 2	Q9UQB8	BAIAP2
Cystatin-A	P01040	CSTA
Platelet endothelial aggregation receptor 1	Q5VY43	PEAR1
Deoxyribonuclease-2-alpha	O00115	DNASE2
Calumenin	O43852	CALU
Galectin-7	P47929	LGALS7
GTP-binding nuclear protein Ran	P62826	RAN
Cofilin-1	P23528	CFL1
Cofilin-2	Q9Y281	CFL2
Peripherin	P41219	PRPH
Granulins	P28799	GRN
Galectin-1	P09382	LGALS1
Transgelin-2	P37802	TAGLN2
Vimentin	P08670	VIM
Basement membrane-specific heparan sulfate proteoglycan core protein	P98160	HSPG2
